# Development and validation of elder-friendly urban spaces questionnaire (EFUSQ)

**DOI:** 10.1186/s12877-019-1355-0

**Published:** 2019-12-02

**Authors:** Azadeh Lak, Reihaneh Aghamolaei, Hamid R. Baradaran, Phyo K. Myint

**Affiliations:** 1 Faculty of Architecture and Urban Planning, Shahid Beheshti University, Tehran, Iran; 2 Faculty of Fine Arts, University of Tehran, Tehran, Iran; 3 Department of Epidemiology, School of Public Health, Iran University of Medical Sciences, Tehran, Iran; 4 Ageing Clinical & Experimental Research Team, Institute of Applied Health Sciences, University of Aberdeen, Aberdeen, Scotland UK

**Keywords:** Older people, Public space, Age-friendly environment, Active aging

## Abstract

**Background:**

Considering the lack of specific measurement tools to study elders’ perceptions in outdoor spaces, the study objectives were to derive and validate a questionnaire that assesses the essential features of elderly-friendly urban spaces.

**Methods:**

We used closed-ended questions in two phases. In the first qualitative phase, a preliminary questionnaire was defined using grounded theory. In the second phase, the psychometric properties of the elderly-friendly urban spaces were examined through validity and reliability indices.

**Results:**

The findings of the first phase led to a preliminary item extraction and questionnaire with 15 major domains based on three dimensions: place function, place preferences, and process. In the second phase, a 48-item questionnaire, based on three dimensions, in addition to personal characteristics, was introduced.

**Conclusions:**

The Elderly-Friendly Urban Spaces Questionnaire (EFUSQ) can be adopted in various communities in understanding of how to create age-friendly urban spaces to promote active aging.

## Background

The population of older people aged ≥65 years is predicted to grow from 524 million in 2010 to approximately 1.5 billion by 2050 in an exponential trend golbally [1]. A high percentage of this drastic growth is expected to occur in urban areas [2]. Studies have revealed the influence of the environment on older peoples’ health, physical activity, and well-being at the neighborhood and public space scales. Hence recent attention has been paid in making public spaces suitable for the active aging and aging in place of the population [3].

Open and green spaces provide social interaction opportunities and generate a sense of community. They also promote social engagement, physical activity, relaxation, and interaction with nature [4]. These places are accessible most of the time for the majority of the public with low cost [5]. Research is needed to create valid and reliable tools for assessing age-friendliness of urban places to be used at baseline and follow-up so as to be able to evaluate improvements over time [6]. However, a considerable gap exists between research run on age-friendly assessment methods and the evolving local community initiatives [6]. Age-friendly studies highlight the importance of local surveys to precisely obtain information and incorporate them into local attributes through the application of grounded approaches [7].

It is, therefore, essential to develop population-specific tools to collect information on older people’s expectation of public spaces. The objective of this study is to develop and determine the psychometric properties of a tool for measuring age-friendly urban spaces according to older people’s preferences. This step is a critical prerequisite for developing age-friendly urban spaces to promote active aging cities.

## Methods

### Theoretical background

According to the guidelines introduced by WHO, an age-friendly city encourages active aging by optimizing opportunities for health, participation, and security to enhance the quality of life [8]. WHO has proposed 6 determinants for the concept of active aging in cities: [1] health and social services, [2] behavioral, [3] personal, [4] physical environment, [5] social, and [6] economic determinants [9]. “Active aging” is perceived as the desire and ability of older people to integrate physical activity into their daily routines and engagement in economic and socially productive activities [10].

There are many different methods to assess the age-friendliness of urban spaces [6]. Current methods of assessing older peoples’ view of the built environment can be categorized into 3 groups. Observational audit tools typically aimed to capture descriptive and objective data on specific street-level attributes such as presence and qualities. The second method is a well-established tradition of perceived-environment measures through surveys to collect self-reported data [11, 12]. Lastly, spatial qualitative methods use a more heterogeneous group of tools, comprising techniques such as photo-voice, walk-along interviews, or virtual reality experiments, as exemplified in a recent review of qualitative studies [11, 12].

**Fig. 1:**
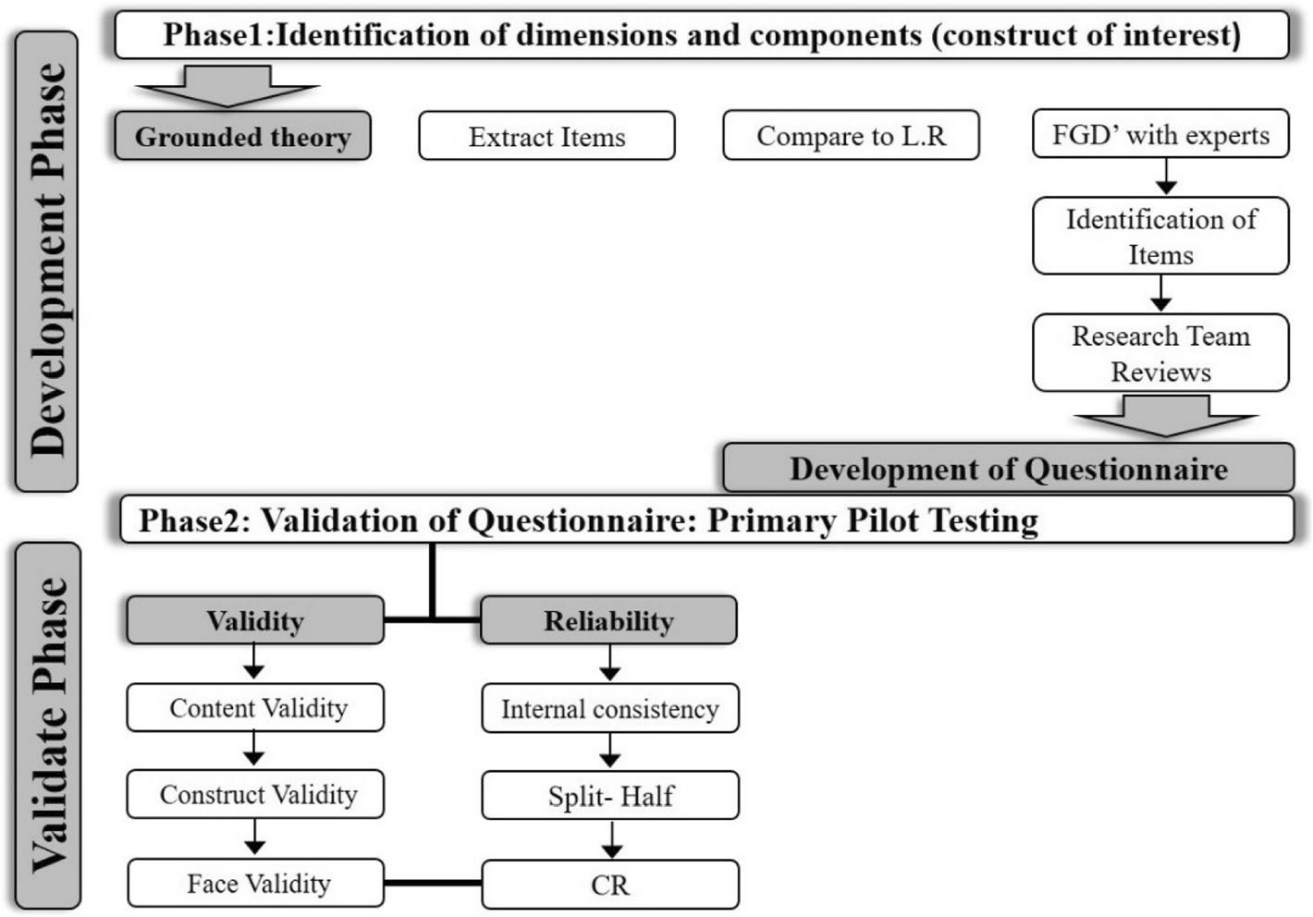
This diagram shows the process of questionnaire development including identification and validation phases

The objective of this study was to develop and determine the psychometric properties of the developed questionnaire for measuring age-friendly urban spaces according to older peoples’ preferences. Developing the questionnaire and its validation is done in two phases (Fig. 1).

The objective of the first phase was to develop the overall scheme of the questionnaire based on grounded theory (GT) and context characteristics. The extraction and design of the items and phrases of the initial questionnaire consisted of three steps: [1] adopting the GT (qualitative research and extracting appropriate phrases through content analysis technique), [2] conducting desk study and extracting the phrases and [3] designing the initial questionnaire.

The objective of the second phase was to validate the questionnaire developed in phase 1 by assessing the validity of the psychometric characteristics of the questionnaire and assessing reliability through structural validity, split-half analysis, and Cronbach’s α coefficient in SPSS 22. Validity analysis was checked by 3 indicators of content, construct, and face validation according to Waltz and Bausell content validity index and Lawshe content validity ratio [13, 14]. The study protocol was approved by the Ethics Board of Iran University of Medical Sciences.

### Grounded theory (GT) and item extraction

The purposeful sampling is used to have maximum variation in the age, sex, literacy, physical and mental health status, and socioeconomic status with a high presence in neighborhoods’ community centers with registered local information in the health department of the community center in Tehran’s neighborhoods. Since the participants in the GT study were selected from older people living in Tehran. The inclusion criteria were [1] age over 65 years, [2] local residents in neighborhoods, [3] willing to participate in the study and [4] providing consent.

The interviews were carried out with 54 older participants who were presents in urban outdoors 3–5 times a week. They were chosen from different public spaces such as parks, streets, and squares in different neighborhoods with different socio-economic classes which have active community centres to collect the elders’ health information from June and July 2018 (Table 1). The duration of the interviews was 20 to 45 min depending on the participant’s level of interest and cooperation (Table 1).

**Table 1:** The participants’ socio-demographic status who attended the interview

Feature	Participants (*n* = 54)
Age group	65–75: 28
	75–85:26
Gender	29 F
	25 M
Education level	Undergraduate: 19
	Graduate:18
	Postgraduate:17
General Health perception (self – reported)	Good Health: 24
	Moderate Health:12
	Poor Health:18
Socio- economic status	Middle- High:28
	Poor- low:26

Moreover, a Focus Group Discussion (FGD) with 12 older peoples (7 women and 5 men) among interviewees was held for trustworthiness in the City Council of District 10 in Tehran Municipality in August 2018.

During the semi-structured interviews according (Table 12), the participants were asked the following questions: How do you like this place to be? What qualities should this place have so that you would want to spend more time in it? The subsequent questions were asked according to the participants’ responses to these two initial open-ended questions. The data were analyzed using Strauss and Corbin’s coding supervised method by two people in the research team’s experts [15]. The last five interviews and the FGD were conducted after reaching theoretical saturation for more certainty and validity.

The credibility of data was assured through peer checking and member checking [16–18]. Peer checks were conducted via weekly research team meetings during which the emerging data were discussed and reviewed and analyzed the data among research group. Member checks occurred by providing a summary of the analyzed interviews and extracted codes to participants so the research team could be asked and incorporated their feedback and ideas for corrections. In addition, the quality of public places was appraised through observational field studies by applying the urban design techniques to assess public spaces’ qualities for instance Jan Gehl’s toolbox [19]. Thus, conformability was observed by considering the opinions of other researchers and transferability by fully describing all the stages of the procedure [18].

### Item finalization

The relevant literature was reviewed to validate the extracted subcategories. In this process, all of the extracted codes are assessed by similar concepts in the literature of this domain (Table 13).

The extracted items and gathered data from desk study are used to guide item development. The developed questionnaire consisted of three scales: place (functional dimension), place (preferred dimension) and process (environments). All items used in the questionnaire were locally experienced items by the elder (Table 2).

**Table 2:** The extracted items from GT and literature reviewed during phase 1

Domains/ Categories	Scales/ Subcategories
Personal characteristics (socioeconomic status)	Age
	Gender
	Marital status
	Occupation
Place (functional dimension)	Density
	Amenities (Access to services)
	Safety (Traffic)
	Aesthetics (design)
	Landscaping
	Comfort
	Environmental cleanness (Visual, air, noise, pollution)
Place (preferred dimension)	Security (Crime)
	Security (Fear of falling)
	Security (Fear of losing/ wayfinding)
	Aesthetics (experienced environment)
Process (environments)	Social environment
	Cultural environment
	Sense of belonging
	Life satisfaction

The questionnaire was initially designed in the Persian language and then checked by two experts in Persian literature to assure cultural appropriateness. In addition, the questionnaire was piloted on a group of 18 older people, and modifications were made prior to the study.

As an initial instrument, the questionnaire of the frequency of use was devised based on a 5-point Likert-type scale (almost always, often, sometimes, seldom, and never) (Table 3). The reasons for selecting this scale were its pivotal role in building the older peoples’ preferences in public spaces and its focus on dynamic interactions between people and the environment [20].

**Table 3:** The scales, items, and the number of items presented in the questionnaire

Domains	Scales	Number of items
PF: Place (functional dimension)	Density	9
	Amenities (Access to services)	10,11,12,13,14
	Safety (Traffic)	15,16
	Aesthetics (Objective)	26,27
	Landscaping	30,31,32
	Comfort	33,34,35,36
	Environmental cleanness (Visual, air, noise, pollution)	37,38
PP: Place (preferred dimension)	Security (Crime)	17,18,19
	Security (Fear of falling)	20,21,22,23
	Security (Fear of losing/ wayfinding)	24,25
	Aesthetics (Subjective)	28,29
PE: Process (environments)	Social environment	39,40,41,42
	Cultural environment	43,44
	Sense of belonging	45,46,47,48
	Life satisfaction	49

### Questionnaire validation

After pilot testing and revisions of the questionnaire, a second pilot test was run on the intended respondents for initial validation among 42 elder people participated in the qualitative phase. After considering validity and reliability, the final version of the questionnaire was given to the specified sample of 350 respondents in two neighborhoods.

### Questionnaires’ validity

In this section three concepts of content, face, and construct validity are considered to investigate the questionnaire validity.

### Content validity

Lawshe’s method was adopted for content validity analysis by calculating the Content Validity Ratio (CVR) [14]. The questionnaire items were evaluated by a group of nine experts in landscape architecture, urban design, planning, and gerontology. The experts rated items either as *essential*, *useful*, or *not necessary*. A dichotomy was then devised from the 3-point rating scale into *essential*, *useful*, and *not necessary*. The revised binomial probability distribution for Lawshe’s critical values was applied in excluded items rated as *not necessary* [21]. A scale content validity index (S-CVI) was calculated for each scale by averaging the CVR for all the retained items in the scale [22, 23]. If CVI is higher than 0.9, it indicates excellent content validity at the scale level [22].

### Face validity

Initially, 18 older people were asked whether there was any ambiguity in items of the questionnaire, and if any, the items were modified. In the quantitative phase, the impact score (frequency in importance) was evaluated by nine experts considering difficulty, inappropriateness, and ambiguity of the phrases. Qualitative face validity was determined by a panel including three urban designers, three urban planners, two gerontologists and one epidemiologist. These specialists evaluated the level of difficulty, inappropriateness, and ambiguity of the phrases. Their comments were used in the questionnaire.

The impact score was calculated for each question to determine the quantitative face validity (Eq. 1) [24]. For each of the 41 questions, a 5-point Likert scale was used to determine impact score. This scale range included strongly agree (score 5), agree (score 4), no idea (score 3), disagree (point 2), and strongly disagree (score 1). After completing the questionnaire by the target group (by 12 participants of FGD and 9 health expert), the face validity of the item was calculated by using the impact score equation (Eq. 1). The impact scores equal to or greater than 1.5 are considered appropriate [25].

1 $$ \mathrm{Impact}\ \mathrm{Score}=\mathrm{Frequency}\ \left(\%\right)\times \mathrm{Importance}\ \mathrm{value} $$

### Construct validity

To examine the construct validity and internal consistency of the final questionnaire, a random sample of 350 older people (≥ 65 years old) from different public spaces in the selected district was invited to participate in answering the questionnaire in August and September of 2018. Stratified random sampling is used in this study to improve the representative ness of the sample. The population of the elders is divided into nine neighborhoods with different public spaces called sub-region and random samples are drawn from each of these public spaces (parks, community centres) in sub-regions. The time needed to complete the questionnaire was 30–40 min. Construct validity was determined by the Kaiser–Meyer– Olkin (KMO) value. The Bartlett’s test of sphericity was used to test the sampling adequacy and the strength of correlations between each scale item, respectively [26].

We applied Partial least squares (PLS) to test the conceptual model. PLS is useful in structural equation modeling for applied research projects, especially when the participants are limited with skewed data distribution [27]. To measure the validity in PLS, the 3 indicators of Average Variance Extracted (AVE), Confirmatory Factor Analysis (CFA), and Fornel and Larker methods were adopted [28]. Fornel and Larker introduced the AVE criterion in 1981 to measure convergent validity and claimed that the critical number is 0.5. Any output of more than 0.5 indicates acceptable convergence [28]. The AVE criterion indicates the shared average variance between any structures and the indices thereof, and the more the correlation, the greater the goodness-of-fit. Convergent validity was applied as the substantial criterion as the goodness-of-fit measuring model in PLS.

### Questionnaires’ reliability

We evaluated the reliability of the questionnaire through internal consistent split-half reliability, composite reliability (CR), and item reliability.

### Split-half & internal consistency

The split-half method as an improvement method is used when it may not be possible to use the same test twice and to get an equivalent form of test especially among older adults [29]. The items of a test were divided into two matched halves and, then, the score of the first half questions and that of the second half are calculated [30]. The split-half method cannot be applied with heterogeneous questionnaires, as the division of the questionnaire will not yield equivalent forms. In this situation (heterogeneous questionnaires), one may repeat questions throughout the questionnaire, while only the original question is kept in the final form [30].

In this study to divide the measuring instrument into two halves, the correlation coefficient was calculated between scores of odd numbered and even numbered items based on Eq. 2. Coefficient α represents the average of all possible split-half estimates.

2 $$ \mathrm{Reliability}\ \mathrm{coefficient}=\left(\mathrm{Correlation}\ \mathrm{Coefficient}\ast 2\right)/\left(\mathrm{Correlation}\ \mathrm{Coefficient}+1\right) $$

### Composite reliability (CR)

A more up-to-date PLS criterion named “composite reliability” is applied in relation to coefficient α, as this criterion is introduced in1974 [31]. Here, the validity is measured in accordance with the correlations within, not in an absolute sense. Accordingly, both of these criteria are applied to measure validity in PLS more accurately. In case the CR volume for each structure is higher than 0.7, appropriate internal stability is assured for the measuring methods [32].

### Item reliability (factor loading)

Factor loading is calculated through analyzing the correlation values of a structures’ indices in PLS. The obtained volume ≥ 0.5 indicates that the variance between the structure and its indices are greater than its measuring error variance and that the validity of the measuring model is acceptable [33].

## Results

### Questionnaires development

In the first step, participants’ objective and subjective preferences were considered in a psychological process. Statements describing the preferences of older people were extracted from the interviews. At the initial stage, a total of 98 statements were extracted. After assessing contextual overlapping and closeness, they were reduced to 65 concepts, 15 subcategories, and three categories (Table 2 and Table 13).

In the next step, the related terms were searched in Google Scholar, Science Direct, Sage, Wiley online, Springer, and Scopus. In total, in this context, 25 measuring tools were found while “Age-friendly Cities Checklist of Essential Features” and “AARP Livable Communities” had the most appropriate statements [8]. From these two questionnaires, eight appropriate concepts corresponding to the extracted qualities of the subcomponents were extracted.

Then, we combined all as 73 concepts (65 from the interviews and 8 from the literature review) and were assessed again for closeness, similarities, and relativeness. Factors with conceptual similarity and overlaps were eliminated, reducing the concepts to 40 statements. The environmental properties of the older people were categorized and the questionnaire with a Likert-type scale response was constructed as follows:

- Statements in the first person singular, with a true and false response range. For instance, the signs and the buildings’ façade in the neighborhood assist me to find my way (strongly disagree, somewhat disagree, neither agree nor disagree, somewhat agree, strongly agree).
- Statements in the first person singular, with a range from none to many. For example, the path on the sidewalk from my home to the bus/ subway is comfortable (always, very frequently, rarely, very rarely, never).
- Statements in second person singular such as the possibility of seeing friends per week (very high, above average, average, below average, and very low).
- Question statements such as how clean is the air and is it good for taking a walk? (Excellent, above average, average, below average, and very poor).

Finally, a 5-scale questionnaire was developed to assess and validate the temporal stability (always, very frequently, rarely, very rarely, and never) (Table 14).

### Questionnaire’s validation

#### Demographic variables analysis

A total of randomly selected 350 older people from public spaces of Tehran 10th municipality region. This region is claimed partially as the highest populated region with the most the elder population in Tehran. According to the low area of residential settelments in this region, the majority of the older adults use neighborhoods’ public spaces [34].

Their mean (SD) age was 76.3 ± 9.2 years, and 61.3% of the total participants were male, 73.5% were married, and 27.2% had not finished high school. Table 4 showes the summary of participants’ demographic information for the questionnaire validation phase.

**Table 4:** The participants’ demographic information in quantitative phase

	Participants (*n* = 350)	
	Variables	*N* (%)
Gender	Male	252 (61.3%)
	Female	157 (38.4%)
Marital status	Single	17 (4.1%)
	Widow	91 (22.1%)
	Married	302 (73.5%)
Education	No literacy	143 (34.8%)
	Lower of diploma	153 (37.2%)
	Diploma	84 (20.4%)
	Academic	26 (6.3%)
Occupation	Employed	50 (12.1%)
	Housewife	122 (29.7%)
	Retired	238 (57.9%)

### Questionnaires’ validity

#### Content validity and face validity

The Lawshe method of content validation was used to validate the questionnaire and showed the content index and validity ratio of 0.82 and 0.79, respectively. According to Lawshe, the minimum acceptable CVR is 0.78 and CVI ≥0.82 [14]. However, if a question has a value < 0.78 and the mean of judgments > 1.50, it is acceptable. Moreover, face validity with the impact score of 1.8 is considered appropriate. Table 5 indicates the content validity of domains used in the questionnaire. Construct Validity:

**Table 5:** The content validity of domains used in the questionnaire

Domains	CVI (%)	CVR (%)
Density	0.81	0.73
Amenities (Access to services)	0.71	0.69
Safety (Traffic)	0.7	0.78
Aesthetics (Design)	0.82	0.89
Landscaping	0.89	0.76
Comfort	0.9	0.96
Environmental cleanness (Visual, air, noise, pollution)	0.95	0.98
Security (Crime)	0.92	0.93
Security (Fear of falling)	0.95	0.94
Security (Fear of losing/ wayfinding)	0.75	0.78
Aesthetics (Subjective)	0.79	0.75
Social environment	0.79	0.75
Cultural environment	0.92	0.70
Sense of belonging	0.82	0.70
Life satisfaction	0.83	0.70

The construct validity of all the respondents was analyzed using CFA. To extract the underlying factors, the principal component analysis was run through varimax rotation. The sampling adequacy and sphericity were tested using KMO and Bartlett’s test, respectively. The findings indicated strong significance for Bartlett’s test (x = 9951 and *p* < 0.001). Moreover, the KMO value was measured to be 0.88, indicating that the correlations among the items of each scale were sufficiently strong for the factor analysis [26, 35].

The AVE and Fornel and Larker methods were applied to measure validity, and the findings are presented in Table 6. In this study, the AVE for all variables was more than 0.5 (Table *6*)*,* which showed the c*onvergent validity (CV)* [26, 35].

**Table 6:** The validity of AWE on older people preferences and place attributes

Variables	AVE
Older people preferences	0.7844
Place	0.8240

As observed in Tables 7 and 8, all relationships were statistically significant because of their absolute value, which was less than 1.69. The factor loadings and the path coefficients, > 0.4, showed that the analyzed variables had acceptable validity (Tables 7 and 8).

**Table 7:** The factor loading calculated for the subcategories of PF, PP, and PE

Older peoples’ preferences		Factor Loading
Place Function (PF)	Density	0.8849
	Amenities (Access to services)	0.8864
	Safety (Traffic)	0.5679
	Aesthetics (Objective)	0.5938
	Landscaping	0.7277
	Comfort	0.6552
	Environmental cleanness (Visual, air, noise, pollution)	0.6903
Place Preferences (PP)	Security (Crime)	−0.2212
	Security (Fear of falling)	0.6091
	Security (Fear of losing/ wayfinding)	0.3721
	Aesthetics (Subjective)	0.5368
Place Environment (PE)	Social environment	0.743
	Cultural environment	0.7908
	Sense of belonging	0.4175
	Life satisfaction	0.5111

**Table 8:** The results of factor loadings and path coefficient for the place and older people preferences and three dimensions of PF, PP, and PE

	Path coefficients (factor loading)
PLACE - > Older peoples’ preferences	0.548
PF - > Older peoples’ preferences	0.315
PF - > Place	0.576
PE - > Older peoples’ preferences	0.578
PP - > Older peoples’ preferences	0.276
PP - > Place	0.503

The third method for assessing validity is Fornell - Larker’s method, which analyzes convergence validity. Results showed that the AVE value for the main matrix diameter was more than its lower number of the main dimension, thus convergent validity was confirmed [28] (Table 9).

**Table 9:** The discriminant validity of Fornell-Larcker test for the main domain of the questionnaire

	Older people preferences	Place	PF	PE	PP
Older people preferences	1	0	0	0	0
Place	0.8753	1	0	0	0
PF	0.8639	0.9272	1	0	0
PP	0.7414	0.9052	0.697	0.4703	1
PE	0.8887	0.5655	0.5767	1	0

### Questionnaires’ reliability

The composite reliability (CR) was measured in PLS. Results showed that the Cronbach’s alpha was 0.81, the Spearman-Brown coefficient 0.72, and the Guttman split-half coefficient 0.73, suggesting high stability and internal consistency of the items. Table 10 showes the the validate Cronbach alpha for each item. Moreover, Table 11 indicates the validate composite reliability (CR) for both Older people preferences and Place in developed questionnaire. In this context, scores were calculated and the correlation between scores for both measurement times was determined using the Spearman correlation coefficient, revealing 0.85 at *p* < 0.001. As the observed results were less than 0.7, thus appropriate stability was approved for all variables.

**Table 10:** The Cronbach Alpha for the extracted dimensions

Dimensions	Domains	Cronbach alpha (%)
PF (function)	Density	0.88
	Amenities (Access to services)	0.87
	Safety	0.75
	Aesthetic	0.79
	Landscape	0.87
	Comfort	0.91
	Cleanness	0.95
PP (preference)	Security (crime)	0.87
	Security (Fear of falling)	0.87
	Security (Fear of getting lost)	0.88
	Aesthetic (Image)	0.84
PE (environment)	Social Environment	0.78
	Cultural Environment	0.90
	Sense of Belonging	0.90
	Life Satisfaction	0.81

**Table 11-:** Reliability indicator tested for older people preferences and place attributes

Variables	Cronbach’s α	CR
Older people preferences	0.725	.879
Place	0.786	.903

## Discussion

This study reported the development and validation of an older people-friendly public space tool as a measure based on the perceived and preferred outdoor urban environment in a special context. This type of instrument fills important research and implementation gaps to define the older people needs and expectations of active living. The study highlighted that this developed tool would be suitable for the assessment of public spaces based on adults’ preferences.

Results of the present study indicate that public spaces evaluation scale incorporate density, amenities (access to services), safety aesthetic (design), landscaping, comfort, cleanness, security (from crime), security (fear of falling), security (fear of getting lost), aesthetic (image), social environment, cultural environment, sense of belonging, and life satisfaction. These indicators are useful in assessing the older peoples’ perception of age-friendly environments in urban neighborhoods in Tehran.

Public places are important for older people’s health and thus it is important to understand which aspects of built and social environments are essential in improving the use of public spaces with the view of promoting active aging and aging in place. Creation of age-friendly active living cities has increasingly been recognized as an important health policy strategy and require robust new methods that are suitable for intersectoral actions and transdisciplinary approaches [36]. Implementation of such methods promotes the participation of adults in public spaces and their involvement in urban planning and design [37].

The scales and dimensions for all the constructs measured in the questionnaire met the standard criteria for excellent content validity [22]. CVR and CVI validity indices were in line with the existing literature [38]. The results of construct validity revealed an appropriate correlation between extracted items; however, the multidimensionality of different scales was observed. The observed dimensions or subscales were in parallel with the content of the urban design guidelines examined. The experiences about density, amenities (access to services), safety (traffic), aesthetics (design), landscaping, comfort, and environmental cleanness (visual, air, noise, pollution) were measured through place function dimension. Adherence to security (crime), security (fear of falling), security (fear of losing/wayfinding), and aesthetics (image) was evaluated using place preference scale. Social environment, cultural environment, sense of belonging, and life satisfaction were measured using process scale. Also, age, gender, marital status, occupation, and education were measured using person statues scale [38, 39].

The findings are compatible with those of previous studies, as all of those attributes that can compromise the basic qualities of public spaces are partially dependent on characteristics of the physical environment. However, they are also influenced by “soft” aspects of the environment and can significantly add or detract from the incentives and subjective experience of a particular public space. Furthermore, the findings of this study fit well with the 4 main features of Pikora conceptual framework for assessing environmental determinants of active travel functionality, safety, aesthetics, and destinations, and reviews [40].

WHO defines age-friendly outdoor spaces as public spaces that have the following criteria: clean and pleasant; sufficient green spaces and landscape; well-maintained and safe; well-maintained pavements; free of obstructions; non-slip pavements; comfortable for wheelchairs; accessible and safe design for traffic and pedestrians at intersections and pedestrian crossings; street lighting; and police patrols and community education [8]. “Livable Communities: An Evaluation Guide” claims that walkable communities improve active aging. The required indicators are designing high-quality sidewalks and their maintenance, traffic signals, pedestrian amenities, safety and security (lighting, sight Lines, eye/ear isolation, entrapment areas, escape routes, sense of ownership/maintenance, and police services) [41].

Analysis of item-to-total correlation confirmed that each item belonged to its corresponding subscale. The internal consistency analysis with Cronbach’s α revealed an acceptable level of internal consistency for the total scales and subscales identified through factor analysis for PF, PP, and PE domains. Although certain subscales have moderate alpha values, the Cronbach’s α, within 0.5 and 0.8 range, has been reported in the literature [38].

Furthermore, the moderate Cronbach’s α for items in each scale or subscale indicates that items are interrelated with little redundancy [42]. Thus, each item in each scale measures something different. The low inter-item correlation indicates lower homogeneity, which is preferable, particularly for application in areas of motivation and personality, and is the case in this questionnaire [42].

In terms of temporal stability, the scores for all the retained items in the different scales and subscales indicated a level of good to excellent stability [43]. The results for the temporal stability of the current scales corresponded to the reliability results of the age-friendly public spaces of WHO checklist and livable communities [6, 44].

Age-friendly community initiatives have excellent opportunities to combine the advantages of qualitative and quantitative methods to conduct a baseline assessment that is comprehensive and representative of the diverse older adult population. Therefore, this study has provided the first validated psychometric tool for assessing older peoples’ preferences in public spaces as age-friendly public places in Iran. The results indicated that the developed scales are valid and reliable to measure the corresponding constructs on a constant basis.

This tool includes items that are interrelated within each scale or subscale, as measured by Cronbach’s α statistic, with little redundancy. This tool measures the type and level of the likability of public places in the older peoples’ perspective. Further, it can measure the environmental potential to encourage older people to spend more time in outdoor spaces.

In summary, through analyzing older peoples’ experience we have developed tools to measure the possibility and concreteness of age-friendly environment at micro, meso, and macro scales. The extracted components from qualitative studies have led to developing a psychometrical tool to measure the validity and stability of age-friendly public spaces based on the older peoples’ experience fit for local communities. We have shown the robustness of this method by systematically examining the validity and reliability thus such methodology can be adopted in various communities in understanding of how best to create age-friendly urban spaces to promote active aging.

This study, however, has several limitations. First, the questionnaire does not include all the proposed dimensions of the elders’ preferences of desired public spaces because of integration of all dimensions could result in developing an instrument with many items, making it very challenging to be applyed for the seniors. Second, the validity and reliability of the questionnaire were tested in only one region in Tehran as known to comodate the highest rate of older adult in Tehran. Therefore, it strongly advises that the generalization of the findings should be done with caution. Third, the sample size was about 350 peolple which is recommended for more than 480 older people to validate this questionnaire. Finally, most of recruited participants in qualitative phase had high education to be more familiar in interviewing process. More studies are required to refine items and generalize the findings to other industries or organizations. In addition, another limitation of this study is that no bias analysis was performed between the participants.

## Conclusions

It is conluded that the Elderly-Friendly Urban Spaces Questionnaire (EFUSQ) can be adopted in various communities in understanding of how to create age-friendly urban spaces to promote active aging.

## Data Availability

An anonymized dataset is available by request from corresponding author.

## Appendix

**Table 12:** Survey questions in phase 1

1.	Gender
2.	Education level
3.	Income level
4.	Address (as in neighborhood only)
5.	How frequent do you visit public places?
6.	How much time approximately do you spend on each visit?
7.	What makes this place special or likable?? If likable why?
8.	Why do you like this place?
9.	What do you like about this place?
10.	What needs to be improved?
11.	What are the contributions of the visiting public spaces to your life?

**Table 13:** Concepts and Subcategories of Age-Friendly Public Spaces by Interviewees

Category: Place		Category: Process			
Functional Dimension	Preference Dimension	Social Dimension	Cultural Dimension	Sense of Belonging	Life Satisfaction
Low density Proper walking path Sanitary service Closeness to home Easy commute to and from home Appropriate urban furniture Safety traffic signs Establish library for the older people Chess game Indoor space Protection from cold Sporting equipment Safety Security Relatively low population Congestion Need for space to sit Need for a hangout Need for environmental comfort Need for illuminate (Natural or artificial) Need for cleanness Need for tidiness Need for law and order Need for calmness Need to be safe from possible harms due to children’s play Noise pollution (non-traffic) Need to separate older peoples’ zone from the children’s game yards Noise pollution Need for proper pavement, not slippery ‍Civic misdemeanor, improper behavior ‍Civic misdemeanor, motor-bike in the park ‍Civic misdemeanor, having pets in the park Need for security (without a police) Need for security (due to presence of too many rascals) Need for security (too many open access ways) Need for security (its generality) Need for security (for the health of animals in the park) Need for small convenient stores Face lifting the old buildings Need for parking Weakness in service Need for sporting and training equipment Need to reduce the slops Need to disperse the drug addicts Need for arbor Need for traffic control Need to control the Youths	Green space Plant and water Tree Cosines Openness Low enclosure Spatial variety Past memories Traditional design and old moments Sense of richness in hearing Presence of artists, poet, Iranian idioms Public memories (sticking to old names) Need for space aesthetics Legibility Not falling Pavement Security Familiarity No crime No theft No graffiti No incivility Easy to wayfinding Order and symmetric design Good pavement No slippery Surface No nuisance	Social interaction Friends and next of kin Social wealth Social strata (good people) Foreigner hater No street vendors Home space satisfaction	Segregated space for female and male Segregate space for older peoples and house pets	Keep old memories Old habits Friendly relations Social capital Social relations	Sense of home Filling required space Sense of home yard Sense of calm at home

**Table 14:** Older people-Friendly Public Space Questionnaire

1.	Age					
2.	Marital status	Single	widowed	Married		
3.	Education level	Illiterate	lower than High school	Diploma		University
4.	Monthly expenses					
5.	Occupation: 1-Employed, 2- Housewife, 3-Retired					
6.	How long have you lived in this neighborhood?					
7.	Address: Ave., St., Alley?					
8.	Why did you choose this neighborhood for living?					
9.	The congestion in the streets and public spaces prevent my walk.	Never	Rarely	Sometimes	Very often	Always
10.	Access to the store, bank, mosque, pharmacy, clinic from my home is an easy walk.	Never	Rarely	Sometimes	Very often	Always
11.	Access to path to bus-stop and metro-station is easy.	Never	Rarely	Sometimes	Very often	Always
12.	There are sufficient and clean public sanitary services in the green space of the neighborhood.	Never	Rarely	Sometimes	Very often	Always
13.	The neighborhood streets have sufficient sidewalks for pedestrians.	Never	Rarely	Sometimes	Very often	Always
14.	There is only one park and green space close to my house.	Never	Rarely	Sometimes	Very Often	Always
15.	The speed of motor bikes and automobiles in the streets and cross roads of the neighborhood is low, thus walking is safe.	Never	Rarely	Sometimes	Very often	Always
16.	The pedestrian signs, street lines, and lights are helpful.	Never	Rarely	Sometimes	Very often	Always
17.	The streets of the neighborhood are well-lit.	Never	Rarely	Sometimes	Very often	Always
18.	The lighting in the neighborhood’s green space is sufficient.	Never	Rarely	Sometimes	Very often	Always
19.	The public and green space here is safe and there are no drug dealers.	Never	Rarely	Sometimes	Very often	Always
20.	The street and green space sidewalks are smooth with no cracks or holes.	Never	Rarely	Sometimes	Very often	Always
21.	The sidewalks’ slopes are acceptable.	Never	Rarely	Sometimes	Very often	Always
22.	The sidewalks are not slippery, and thus are appropriate and safe.	Never	Rarely	Sometimes	Very often	Always
23.	The width of the sidewalks is appropriate for pedestrians.	Never	Rarely	Sometimes	Very often	Always
24.	The billboards and the façade of the buildings help me to find my way in the neighborhood.	Never	Rarely	Sometimes	Very often	Always
25.	I am familiar with the public spaces here and find my way easily.	Never	Rarely	Sometimes	Very often	Always
26.	The neighborhood’s public green spaces are neat and beautiful.	Never	Rarely	Sometimes	Very often	Always
27.	There are new and beautiful buildings here.	Never	Rarely	Sometimes	Very often	Always
28.	The tall buildings make the neighborhood look boring.	Never	Rarely	Sometimes	Very often	Always
29.	The green spaces are cozy and refreshing.	Never	Rarely	Sometimes	Very often	Always
30.	The streets and green spaces are provided with clean and comfortable urban furniture.	Never	Rarely	Sometimes	Very often	Always
31.	The drinking water units, arbors, and recreational facilities in the public and green spaces are sufficient and accessible.	Never	Rarely	Sometimes	Very often	Always
32.	The green spaces of the neighborhood are full of trees, flowers, and fountains.	Never	Rarely	Sometimes	Very often	Always
33.	The green spaces for children are separate from those of the older peoples.	Never	Rarely	Sometimes	Very often	Always
34.	There are some shady sections in the open space to prevent extreme sunshine and cold.	Never	Rarely	Sometimes	Very often	Always
35.	The municipality maintains the good quality of the greenery in the park, streets, and sidewalks.	Never	Rarely	Sometimes	Very often	Always
36.	The municipality is responsible for maintaining the greenery and furniture in the park.	Never	Rarely	Sometimes	Very often	Always
37.	The air quality and temperature are fair here for taking walks.	Never	Rarely	Sometimes	Very often	Always
38.	The sidewalks and public spaces are clean with no garbage, thus there is no bad odor.	Never	Rarely	Sometimes	Very often	Always
39.	The public green space is the place for meeting friends.	Never	Rarely	Sometimes	Very often	Always
40.	In the public green space people behave in a polite manner.	Never	Rarely	Sometimes	Very often	Always
41.	The neighborhood residents are helpful and assist one another.	Never	Rarely	Sometimes	Very often	Always
42.	People in my neighborhood take part in religious ceremonies.	Never	Rarely	Sometimes	Very often	Always
43.	In the public green space, the house pets like cats and dogs do not disturb older peoples.	Never	Rarely	Sometimes	Very often	Always
44.	Space separation for men and women contribute to more comfort of the older peoples.	Never	Rarely	Sometimes	Very often	Always
45.	I admire my neighborhood and I will not live it.	Never	Rarely	Sometimes	Very often	Always
46.	I have many great memories of this neighborhood.	Never	Rarely	Sometimes	Very often	Always
47.	The green space makes me feel as comfortable as my own back yard.	Never	Rarely	Sometimes	Very often	Always
48.	People participate in protecting and cleaning the public spaces of their neighborhoods.	Never	Rarely	Sometimes	Very often	Always
49.	My home is comfortable to live in with adequate space.	Never	Rarely	Sometimes	Very often	Always

## Abbreviations

AVE: Average variance extracted
CFA: Confirmatory factor analysis
CR: Composite reliability
CVI: Content validity index
CVR: Content validity ratio
FGD: Focus Group Discussion
GT: Grounded Theory
KMO: Kaiser–Meyer– Olkin
PLS: Partial least squares
WHO: World Health Organization
